# Extraction of Essential Oils of* Rosmarinus officinalis* L. by Two Different Methods: Hydrodistillation and Microwave Assisted Hydrodistillation

**DOI:** 10.1155/2019/3659432

**Published:** 2019-04-01

**Authors:** Majda Elyemni, Bouchra Louaste, Imane Nechad, Taha Elkamli, Abdelhak Bouia, Mustapha Taleb, Mahdi Chaouch, Noureddine Eloutassi

**Affiliations:** ^1^Laboratory of Materials Engineering and Environment, Faculty of Sciences Dhar El Mahraz, Sidi Mohammed Ben Abdellah University, BP 30003, Fez, Morocco; ^2^Laboratory of Biotechnology, Faculty of Sciences Dhar El Mahraz, Sidi Mohammed Ben Abdellah University, BP 30003, Fez, Morocco; ^3^Laboratory of Biological Test, Food and Nutritional Transition Team (ETAN), Ibn Tofail University, BP 133, Kenitra, Morocco; ^4^Laboratory of Engineering, Electrochemistry, Modeling and Environment, Faculty of Sciences Dhar El Mahraz, Sidi Mohammed Ben Abdellah University, BP 30003, Fez, Morocco

## Abstract

The extraction of essential oils is generally carried out by two main techniques: azeotropic distillation (hydrodistillation, hydrodiffusion, and steam distillation) and extraction with solvents. However, these traditional methods are a bit expensive, especially since they are extremely energy and solvent consuming. This work consists in studying two methods of extraction of the essential oils of* Rosmarinus officinalis *L.: microwave assisted hydrodistillation (MAH) and Clevenger hydrodistillation (CH). Several parameters have been studied: the extraction time, the yield, and the chemical composition of the essential oils as well as the efficiency and cost of each procedure. The results obtained revealed that microwave-assisted hydrodistillation makes it possible to minimize the extraction time of the essential oils in comparison with conventional hydrodistillation. Thus, the same yield of essential oils is obtained for 20 minutes only with MAH while it takes 180 minutes with CH. In addition, the quality of the essential oil is improved thanks to a 1.14% increase in oxygenates. In conclusion, the MAH method offers significant advantages over conventional hydrodistillation and can therefore replace it on a pilot and industrial scale.

## 1. Introduction

*Rosmarinus officinalis L.*, commonly known as rosemary, is a shrub belonging to the Lamiaceae family and native to the Mediterranean basin[1]. This plant has been widely used in traditional medicine since antiquity, and it has also been used as a food preservative and flavoring agent [2, 3].

Rosemary contains an essential oil to which it owes its interesting properties. It is known for its antioxidant, antimicrobial, anti-inflammatory, anticarcinogenic, antidiabetic [4], antinociceptive [5], and antithrombotic properties [6] and antiulcerogens [7], diuretics [8], and hepatoprotective effects [9]. These biological properties have made rosemary a potential new therapeutic agent in the treatment of many diseases. One of the main derivatives of this emblematic plant in traditional medicine is its essential oil.

The essential oil secreted by glandular trichomes is mainly located in leaves and the flowers; the highest quality essential oil is obtained from the leaves [10].

*Rosmarinus officinalis L.* essential oil is usually isolated by hydrodistillation, steam distillation, or extraction with organic solvents. These techniques cause the loss of certain volatile compounds due to long extraction times and degradation of unsaturated or esterified compounds by thermal or hydrolytic effect. For example, monoterpenes may be susceptible to chemical changes under stream distillation conditions and even the conventional solvent extraction during removal of solvent by distillation. In addition, many of these methods are time-consuming and energy intensive [10, 11].

However, in order to reduce the extraction time and improve the quality of essential oils, new extraction techniques have been developed such as microwave assisted extraction, solvent extraction under pressure, supercritical fluid extraction, and ultrasound-assisted extraction [12, 13]. Microwave-assisted hydrodistillation has been used for the extraction of laurel essential oils [14], lavender [15], and thyme [16], and rosemary has also been studied [17, 18]. Faced with all these innovative methods of extraction of essential oils, the choice of the most efficient method is relevant for a better optimization of time, yield, and cost of production.

This work aims to make a comparative study of two methods of extraction of essential oils of Moroccan* Rosmarinus officinalis L*.: conventional hydrodistillation and microwave assisted hydrodistillation. These two methods were chosen to study the effect of microwave energy on the quantity and quality of rosemary essential oil. In addition, the cost, energy consumption, and environmental impact have been optimized in order to have an optimal method for the production of essential oils of better quality, at lower cost, and with good performance and meeting the requirements of the companies.

## 2. Materials and Methods

### 2.1. Plant Material

The samples of rosemary were harvested at the flowering stage during the month of May 2018 in the region of Fez (406 m, 34°01′59′′ Latitude North and 5°00′01′′ Longitude West). Only the aerial part of plant was used; the leaves and the apical parts were dried in the shade for eight days at a temperature room fixed at 25°C.

### 2.2. Microwave-Assisted Hydrodistillation

**Figure 1:**
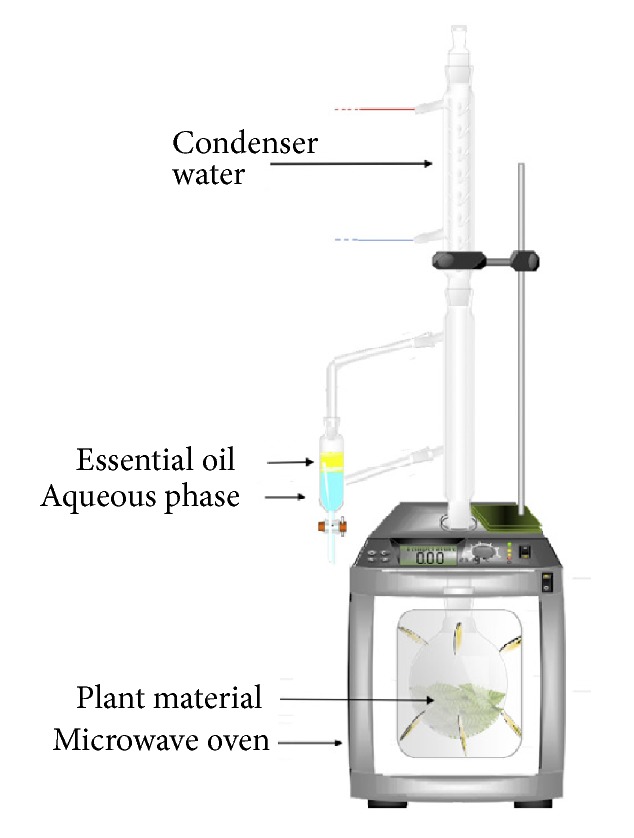
Schematic representation of the microwave-Clevenger.

The microwave-assisted hydrodistillation was carried out using an assembly consisting of a domestic microwave oven (MWD 119 WH, whirlpool, China, 20L, 1100 W), directly connected to a Clevenger-type extractor and a cooling system to condense the distillate continuously. The excess of Condensed water was refluxed to the extraction flask in order to restore the water to the plant material (Figure 1).

Microwave assisted hydrodistillation was carried out under the optimum conditions of the extracting time, microwave power, and ratio water/plant material [19].

100 g of rosemary samples was placed in a 2-liter flask containing distilled water (200 ml), heated inside the microwave oven cavity, and the mixture was heated at a fixed power of 600 W until extraction of the all essential oils.

The essential oils taken from different extractions are dried under anhydrous sodium sulphate and stored in the dark until they are used for analysis. The extractions were done at least three times and mean values of the yield and standard deviation were determined.

### 2.3. Hydrodistillation by Clevenger

For the extraction of essential oils from rosemary by hydrodistillation under optimal operating conditions, a quantity of 100 g of rosemary was added to 800 ml of distilled water in a 2-liter flask [20]. The set was placed in a balloon heater attached to a refrigerator to ensure condensation of essential oils for 3 hours. At the end of the distillation, two phases were observed, an aqueous phase (aromatic water) and an organic phase (essential oil), less dense than water. The essential oil was collected, dried under anhydrous sodium sulphate, and stored in sealed vials in the dark, at 4°C, until used. Experiments were conducted twice for each condition.

### 2.4. Yield of Essential Oils

The yields of essential oil of rosemary were expressed in g relative to 100 g of dry vegetable matter; it was calculated according to Equation (1):

(1) Yield  (%)=Amount  of  extracted  oil  (g)Amount  of  dry  vegetal  matter  mass  (g)×100

### 2.5. Energy Consumption

The energy consumption required to carry out the CH and MAH extractions was determined by a watt-meter connected to the input of the microwave generator and that of the heater.

### 2.6. Quantity of CO2

The carbon dioxide released into the atmosphere is calculated according to the literature: to obtain 1 kWh of coal or other fossil fuels, 800 g of CO2 will be released into the atmosphere during combustion [21].

### 2.7. Chromatographic Analyses of Essential Oils

The chemical composition of the rosemary essential oils extracted by both methods is performed by gas chromatography coupled with mass spectrometry (GC/MS).

The GC analysis was performed using a chromatography equipped with a flame ionization detector (FID) and two capillary columns of different polarities OV type: 101 (25 m x 0.22 mm x 0.25 mm) and Carbowax 20 M (25 m x 0.22 mm x 0.25 *μ*m). The carrier gas is helium with a flow rate of 0.8 ml/min and the oven programming temperature is between 50 and 200°C with a gradient of 5°C/min. CPG/MS coupling was performed on a DB1-type fused silica capillary column (25 m x 0.23 mm x 0.25 *μ*m) with helium as a carrier gas and temperature programming identical to that of the GC.

## 3. Results and Discussion

### 3.1. Yield and Extraction Time of Essential Oils

The descriptive statistics of the yield including mean, standard deviation, standard error, maximum, and minimum from the three repetitions were presented in Table 1. The results showed that the same extraction yield was obtained by the two isolation methods which is of the order of 1.35% ± 0.04% with a confidence interval of 95% (p<0.05) (mean ±1.96 standard error).

**Figure 2:**
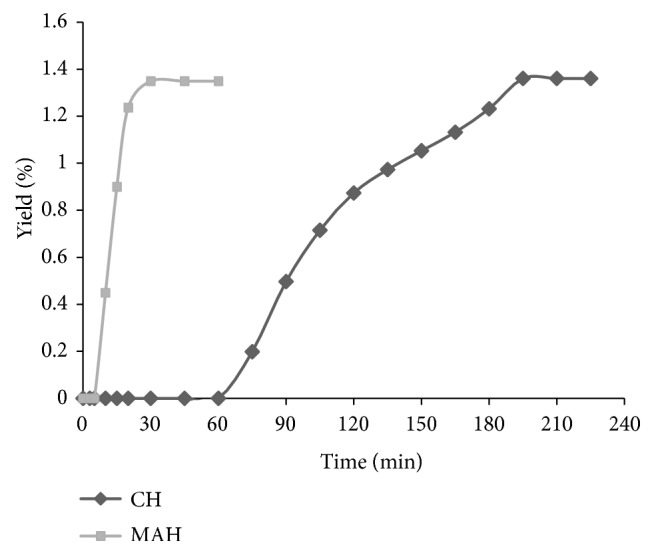
Yield profiles as a function of time for CH and MAH isolations of essential oil from rosemary.

The cumulative yield of the essential oils from rosemary obtained during a single extraction from the three repetitions for each extraction method as a function of time is shown in Figure 2. For both extraction techniques, CH or MAH, the extraction temperature is equal to the boiling of the water at atmospheric pressure (100°C). To reach this temperature and to obtain the distillation of the first droplet of essential oil of rosemary, it is necessary to heat for 3 min only with MAH against 45 min for the CH. A 20-minute extraction time by MAH gives a yield similar to that obtained after 180 min by means of CH.

Several studies have reported that the heat generated by the microwave heating involves a partial pressure gradient of volatile compounds and internal overheating leading to embrittlement or rupture of the cell walls more rapidly and more efficiently [11, 16, 22]. As a result, the kinetics of the extraction process of essential oils is accelerated, which explains the difference in time between the two extraction methods studied. This can be explained by the rate of heat transfer between the two extraction methods. MAHD utilizes three ways of heat transfer within the sample: irradiation, conduction, and convection, while the heat transfer by HD can occur through conduction and convection only.

### 3.2. Chemical Composition of Essential Oils

**Figure 3:**
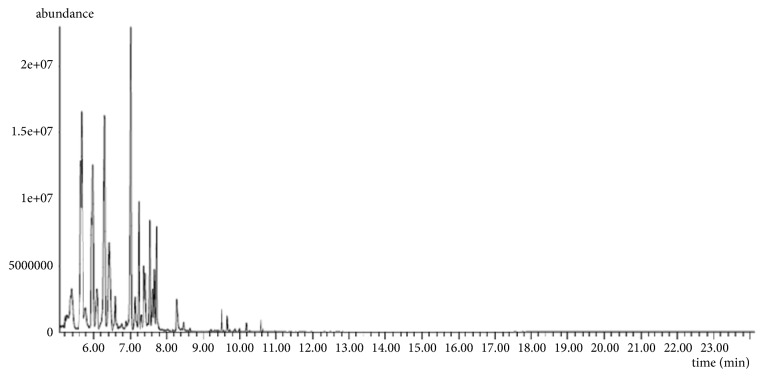
Chromatogram of essential oils of rosemary extracted by CH.

**Figure 4:**
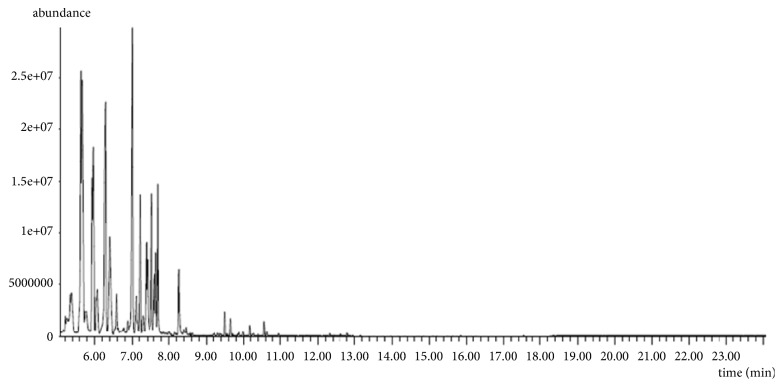
Chromatogram of essential oils of rosemary extracted by MAH.

The results relating to the chemical composition of the essential oils of* Rosmarinus officinalis* L. extracted by the two extraction methods are summarized in Table 2. The chromatographic profiles are illustrated in Figures 3 and 4. These results made it possible to identify 16 compounds for the two methods which represent a total of 99.80% in CH and 99.75% in MAH.

The analysis of the results shows that the chemical composition of the essential oils obtained by the two methods is identical between the two MAH and CH methods with slight quantitative differences in certain constituents. Indeed, the cineole has the major constituent with a slightly higher rate for MAH compared to CH which is, respectively, 32.18% and 31.20%.

However, the percentages of camphor (16.54% in CH and 16.20% in MAH) and *α*-pinene (15.82% in CH and 15.40% in MAH) are lower for MAH compared to those of CH.

A critical observation of the composition of the oils has revealed that the amounts of oxygenated compounds are substantially higher and the amounts of monoterpene hydrocarbons are lower in MAH extracted rosemary oil in comparison with CH.

These results are consistent with those of Bousbia et al. [11], Karakaya et al. [23], and Moradi et al. [18], which confirm that the contents of oxygenated compounds in the oil obtained by MAH are higher than those of the oil obtained by CH. The largest proportion of oxygenates in MAH extracted essential oils is probably due to the low water content in the system and the speed of the heating process compared with conventional hydrodistillation. Thus, the thermal and hydrolytic degradations of oxygenated compounds are limited [24, 25]. Oxygen compounds have a high dipole moment and will interact more vigorously with microwaves and can be extracted more easily unlike monoterpene hydrocarbons that have a weak dipole moment [14].

Oxygen compounds are more valuable than hydrocarbons in terms of their contribution to the fragrance and therapeutic properties of the essential oil and can be used as essential oil quality measures.

### 3.3. Costs, Energy, and Environment

The reduced cost of extraction is clearly advantageous for MAH method in terms of time and energy. The time required for extraction of the essential oils contained in 100 g of rosemary was found at 180 min for the CH and 20 minutes for the MAH, while the energy required to perform this extraction is 2.25 kWh for the CH and 0.23 kWh for the MAH (Table 3). This indicates a substantial saving in the cost of extracting essential oils when using the MAH instead of the HC, in terms of time and energy.

Regarding environmental impact, the amount of carbon dioxide released into the atmosphere during CH (1800 g CO2) extraction is higher than that released during MAH extraction (184 g CO2). Therefore, the MAH represents a "green technology" for the extraction of essential oils.

## 4. Conclusions

The essential oils extracted by MAH are quantitatively (yield) and qualitatively (aromatic profile) similar to those obtained by conventional hydrodistillation, although the treatment time has been significantly reduced in the case of MAH (20 min) by relative to CH (180 min). Microwave-assisted hydrodistillation provides an essential oil with higher amounts of oxygenates substantial energy savings, reduced cost, and reduced environmental burden with less CO2 released into the atmosphere. It can be concluded that the MAH method is a good alternative for extracting essential oils of rosemary.

## Figures and Tables

**Table 1:** Mean value, maximum, minimum, range, Standard error, and Standard deviation of essential oil yield of rosemary.

	Minimum (%)	Maximum (%)	Range (%)	Mean (%)	Standard deviation (%)	Standard error (%)
MAH	0,32	0,39	0,07	0,353	0,035	0,020
CH	0,31	0,37	0,06	0,347	0,032	0,019

**Table 2:** Chemical composition of rosemary essential oils obtained by CH and MAH.

No.	Compounds	Kovat's index	MAH (%)	CH (%)
	*Monoterpene hydrocarbons*		*35,84*	*37,19*
1	*α*-Pinene	939	15,4	15,82
2	Camphene	954	9,16	9,77
3	*β*-Pinene	979	3,72	3,56
4	*α*-Terpinene	1017	2,49	2,44
5	para-Cymene	1025	4,15	4,79
6	Limonene	1028	0,92	0,81
	*Oxygenated monoterpenes*		*63,03*	*61,76*
7	Cineole	1030	32,18	31,2
8	*β*-myrcene	1048	4	3,75
9	Linalool	1097	1,37	1,49
10	Camphor	1146	16,2	16,54
11	Borneol	1169	1,64	1,47
12	*α*-Terpineol	1199	7,36	7,16
13	Verbenone	1205	0,28	0,15
	*Sesquiterpene hydrocarbons*		*0,27*	*0,11*
14	*β*-Caryophyllene	1419	0,12	0,08
15	*α*-Caryophyllene	1423	0,15	0,03
	*Other oxygenated compounds*		*0,61*	*0,74*
16	Bornyl acetate	1289	0,61	0,74
	*Total oxygenated compounds*		*63,64*	*62,5*
	*Total nonoxygenated compounds*		*36,11*	*37,3*
	*Total *		*99,75*	*99,8*

**Table 3:** Energy consumption and CO2 rejected of CH and MAH methods.

	MAH	CH
Extraction time (min)	20	180
Electric consumption (kWh)	0,23	2,25
CO2 rejected (g)	184	1800

## Data Availability

No data were used to support this study.
